# LGI1, CASPR2 and related antibodies: a molecular evolution of the phenotypes

**DOI:** 10.1136/jnnp-2017-315720

**Published:** 2017-10-21

**Authors:** Sophie N M Binks, Christopher J Klein, Patrick Waters, Sean J Pittock, Sarosh R Irani

**Affiliations:** 1 Autoimmune Neurology Group, Nuffield Department of Clinical Neurosciences, John Radcliffe Hospital, Oxford, UK; 2 Department of Neurology, Oxford University Hospitals, John Radcliffe Hospital, Oxford, UK; 3 Department of Neurology, Neuroimmunology Laboratory, Mayo Clinic, Rochester, Minnesota, USA; 4 Department of Neurology, Mayo Clinic, Rochester, Minnesota, USA

**Keywords:** autoimmune encephalitis, epilepsy, limbic system, neuroimmunology, paraneoplastic syndrome

## Abstract

Recent biochemical observations have helped redefine antigenic components within the voltage-gated potassium channel (VGKC) complex. The related autoantibodies may be now divided into likely pathogenic entities, which target the extracellular domains of leucine-rich glioma-inactivated 1 (LGI1) and contactin-associated protein-like 2 (CASPR2), and species that target intracellular neuronal components and are likely non-pathogenic. This distinction has enhanced clinical practice as direct determination of LGI1 and CASPR2 antibodies offers optimal sensitivity and specificity. In this review, we describe and compare the clinical features associated with pathogenic LGI1 and CASPR2 antibodies, illustrate emerging laboratory techniques for antibody determination and describe the immunological mechanisms that may mediate antibody-induced pathology. We highlight marked clinical overlaps between patients with either LGI1 or CASPR2 antibodies that include frequent focal seizures, prominent amnesia, dysautonomia, neuromyotonia and neuropathic pain. Although occurring at differing rates, these commonalities are striking and only faciobrachial dystonic seizures reliably differentiate these two conditions. Furthermore, the coexistence of both LGI1 and CASPR2 antibodies in an individual occurs surprisingly frequently. Patients with either antibody respond well to immunotherapies, although systematic studies are required to determine the magnitude of the effect beyond placebo. Finally, data have suggested that CASPR2 and LGI1 modulation via genetic or autoimmune mechanisms may share common intermediate molecules. Taken together, the biochemical distinction of antigenic targets has led to important clinical advances for patient care. However, the striking syndrome similarities, coexistence of two otherwise rare antibodies and molecular insights suggest the VGKC complex may yet be a common functional effector of antibody action. Hence, we argue for a molecular evolution alongside a clinical and phenotypic re-evaluation.

## Introduction

A key pathophysiological concept in autoimmune neurology is that the neuronal surface autoantibodies (NSAbs), which target the extracellular domains of membrane proteins, have access to their epitope in vivo and can potentially exert a direct effect.^1–3^ In line with this concept, over the past seven years, there has been a molecular evolution in our understanding of antigenic targets within the voltage-gated potassium channel (VGKC) complex, with the identification of human autoantibodies capable of binding either the intracellular or extracellular domains of proteins within the VGKC complex.^4–6^ These biochemical findings, alongside related clinical observations, have refined our understanding of antibody pathogenicity and clinical management.

As optimisation of clinical outcomes is the ultimate goal of this research area, this review is guided by the authors’ extensive clinical experience of patients with leucine-rich glioma-inactivated 1 (LGI1) and contactin-associated protein-like 2 (CASPR2) antibodies and shaped by a patient-centric approach with direct references to patient and family experiences. Within this clinically driven framework, and from the historical starting point of VGKC complex antibodies, we synthesise biochemical and clinical observations to argue for both a molecular divergence in terms of antigenic targets and a clinical convergence in a true spectrum of related diseases with markedly overlapping features. While treatment options will be considered empirically in a field where there are only observational data, available insights into disease mechanisms will guide discussion of future therapies. Throughout, we hypothesise that the field may return to the concept of the VGKC complex as a functional entity through which antibody effector functions may be mediated.

## A molecular–clinical feedback loop: from antigens to patients and back

### Distinct antigenic targets within the VGKC complex

**Figure 1:**
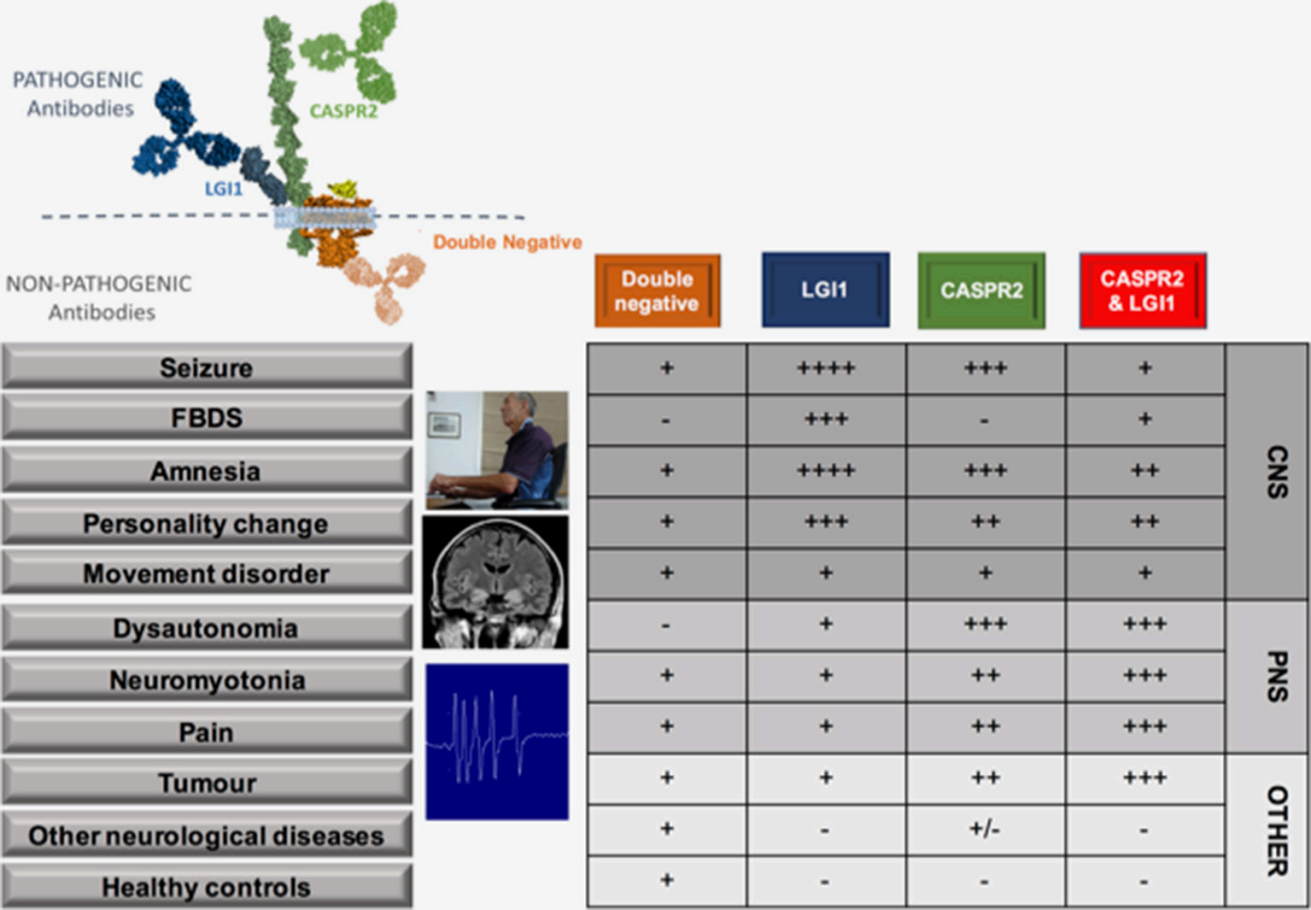
Clinical features of patients with antibodies to LGI1, CASPR2, both LGI1 and CASPR2 and double-negatives. Double-negatives are patients with VGKC complex antibodies, but without LGI1 or CASPR2 reactivities. Figure shows binding of LGI1 and CASPR2 antibodies to the extracellular domains of their respective proteins, whereas the likely intracellular binding of double-negative antibodies, as proven for those that target the intracellular domains of VGKCs themselves.^5^ Relative frequency of various features and conditions in association with each pattern of antibody reactivity denoted by - through ++++. Patient at computer shown during FBDS, medical temporal lobe hyperintensities and neuromyotonic discharges shown. We are grateful to Dr M Symmonds, Oxford, for the neuromyotonic trace. The patient consented to their photograph being used for scientific publications. CASPR2, contactin-associated protein 2; CNS, central nervous system; FBDS, faciobrachial dystonic seizures; LGI1, leucine-rich glioma-inactivated 1; PNS, peripheral nervous system; VGKCs, voltage-gated potassium channels.

Figure 1 depicts known antibodies that target the VGKC complex. VGKC antibodies were originally detected with a radioimmunoassay in which iodinated-alpha dendrotoxin-labelled VGKCs were immunoprecipitated by patient antibodies from solubilised mammalian brain membranes. Therefore, the antibodies were believed to directly target the dendrotoxin-bound VGKCs (Kv 1.1, 1.2 and 1.6).^7^ It is now understood that the patient autoantibodies often target the extracellular domains of VGKC-associated proteins LGI1 and CASPR2, which are tightly complexed with solubilised VGKCs. A rarer subset have been shown to target an in vitro binding partner of CASPR2—contactin-2.^4^ The identification of these specific antigenic targets enabled more detailed classification and dichotomisation of the two molecularly defined syndromes. This approach has facilitated the discovery of some unique clinical characteristics with intriguing pathophysiological implications.^4 8 9^

### A continuous disease spectrum

In sharp contrast to almost all other autoimmune autoantibody-mediated conditions, patients with LGI1 or CASPR2 antibodies are predominantly male, with typical onset in late-middle age. Furthermore, the most common presentation in both is limbic encephalitis,^4 10 11^ with similar MRI changes in the acute phase,^12^ plus extralimbic features including neuromyotonia (NMT), movement disorders, cardiac involvement, sleep disturbance and serum hyponatraemia. While these features occur at statistically significantly different rates in the LGI1 versus CASPR2 antibody patients (figure 1),^4 13 14^ to date, the only absolute clinical distinction is the unique association of faciobrachial dystonic seizures (FBDS) with LGI1 antibodies.^15^ Also, paroxysmal dizziness spells have been described as a LGI1 antibody-specific phenomenon in one paper.^8^ This overlap in clinical features is perhaps even more remarkable given the rarity of these diseases: LGI1 and CASPR2 antibodies found at 1 and 0.3 per million/year in the UK, respectively (Waters and Irani, unpublished observations). Moreover, given the low incidences of these two conditions, coexistence of both LGI1 and CASPR2 antibodies in an individual (‘double-positives’) occurs more often than would be predicted by their independent incidences (table 1). This strongly suggests that the discovery of both antigens in connection with the VGKC complex is more than simply serendipity and that their biology is partially inter-related. A summary of the core features in 46 double-positive patients reported in the literature (table 1) suggests these patients display clinical features associated with both antigenic targets, often with greater weighting towards those more prevalent in CASPR2 antibody disease (figure 1) and a frequent association with an underlying tumour, particularly thymoma.^8 13^ Whether this represents a clue to underlying mechanisms, or reflects reporting bias in the literature towards unusual presentations such as autoimmune neuropathy or Morvan’s syndrome,^8 13^ remains to be determined.

**Table 1:** Features of double-positive patients reported in the literature

**Demographics**	
Age (mean, range)	46.7, 2–86
Male gender, %	28 M, 76% (n=37)
**Clinical presentation**	n (%) with feature*
Morvan’s syndrome	20/37 (54)
Limbic encephalitis	3/38 (8)
Tumour	16/35 (46)
Thymoma	12/34 (35)
Myasthenia gravis	7/25 (28)
Death	3/21 (14)
**Peripheral nerve features**	
NMT/PNH	27/37 (73)
Pain/paraesthesia	21/34 (62)
Peripheral neuropathy	12/31 (39)
**Autonomic features**	
Any dysautonomia	27/32 (84)
Hyperhidrosis	22/30 (73)
Tachycardia/arrhythmia	9/16 (56)
BP abnormalities	7/23 (30)
Sexual dysfunction	1/15 (7)
Gastrointestinal	7/28 (25)
Urinary	5/25 (20)
**Sleep**	
Insomnia	20/28 (71)
**Neuropsychiatric**	
Any	20/23 (87)
Disorientation/confusion/CI	17/38 (45)
Amnesia	7/19 (37)
Hallucinations	12/22 (55)
Agitation/anxiety/depression	10/28 (36)
Delusions	3/18 (17)
**Seizures**	
Any	8/39 (21)
GTCS	5/31 (16)
FBDS/myoclonus	3/31 (10)
Focal/partial	1/31 (3)
Paroxysmal dizziness	1/24 (4)
**Systemic**	
Weight loss	7/17 (41)
Skin lesions/itch/flush	4/16 (25)
Respiratory failure	3/19 (16)
**Investigations**	
Normal brain MRI	16/17 (94)
Normal CSF	6/15 (40)
Serum hyponatraemia	8/29 (28)

Demographic and clinical features of 46 double-positive patients described in the literature from 13 papers. *From the published reports, not all features could be evaluated in all patients, so the denominator varies between features. Additional information, not reported in the publication, was included for the 15 patients with double-positive Morvan’s syndrome in Irani *et al* 13 and may weight this analysis towards features of Morvan’s syndrome.

BP, blood pressure; CSF, cerebrospinal fluid; CI, cognitive impairment; FBDS, faciobrachial dystonic seizures; GTCS, generalised tonic clonic seizure; NMT, neuromyotonia; PNH, peripheral nerve hyperexcitability.

‘Double-negatives’, however, are defined as patients with VGKC complex antibody positivity but without LGI1 or CASPR2 antibody specificities. Recent observations show that almost all double-negative sera show no binding to the surface of live neurons in culture, implying an absence of pathogenic NSAbs.^5^ However, the rodent primary culture system may not express detectable amounts of a surface antigen that may be present in vivo. Therefore, it was important to positively demonstrate that some of these individuals harbour antibodies to intracellular aspects of VGKCs, or even to the non-mammalian dendrotoxin, used as the readout of the assay.^5^ This confirmed the intracellular, likely non-pathogenic, nature of some of the VGKC complex antibodies. While other double-negative targets await discovery, this paradigm is likely to hold true. Even in the few that show binding to the surface of live hippocampal neurons, the target antigen does not appear to be part of the VGKC complex, suggesting two sets of autoantibodies are represented in these sera, and the VGKC complex double-negatives are likely not related to disease pathogenesis.^16^ These observations fit well with the high documented rate of double-negative VGKC complex antibodies in around 5% of healthy controls^17^ and suggest that their presence in non-immune conditions, including structural causes of epilepsy, Parkinson’s and Alzheimer’s disease, may merely reflect this background rate.^5^ Accordingly, in patients with double-negative antibodies and clinically determined encephalitis or autoimmune epilepsy, the treatment should rest on clinical grounds.^18^ Furthermore, this 5% rate in healthy controls somewhat argues against their utility as biomarkers of inflammation, although it remains of immunological interest that double-negative VGKC complex antibodies are very frequently generated in abattoir workers exposed to aerosolised porcine brain, a phenomenon that can be reproduced in experimental mice exposed to aerosolised brain tissue.^19^ Therefore, the VGKC complex remains an immunogenic moiety, but autoantibodies directed against targets other than LGI1 and CASPR2 are increasingly unhelpful clinical tools. Below, we focus on LGI1 and CASPR2 antibodies that are the major pathogenic antibodies within the VGKC complex.

## LGI1 and CASPR2: the clinical features

**Figure 2:**
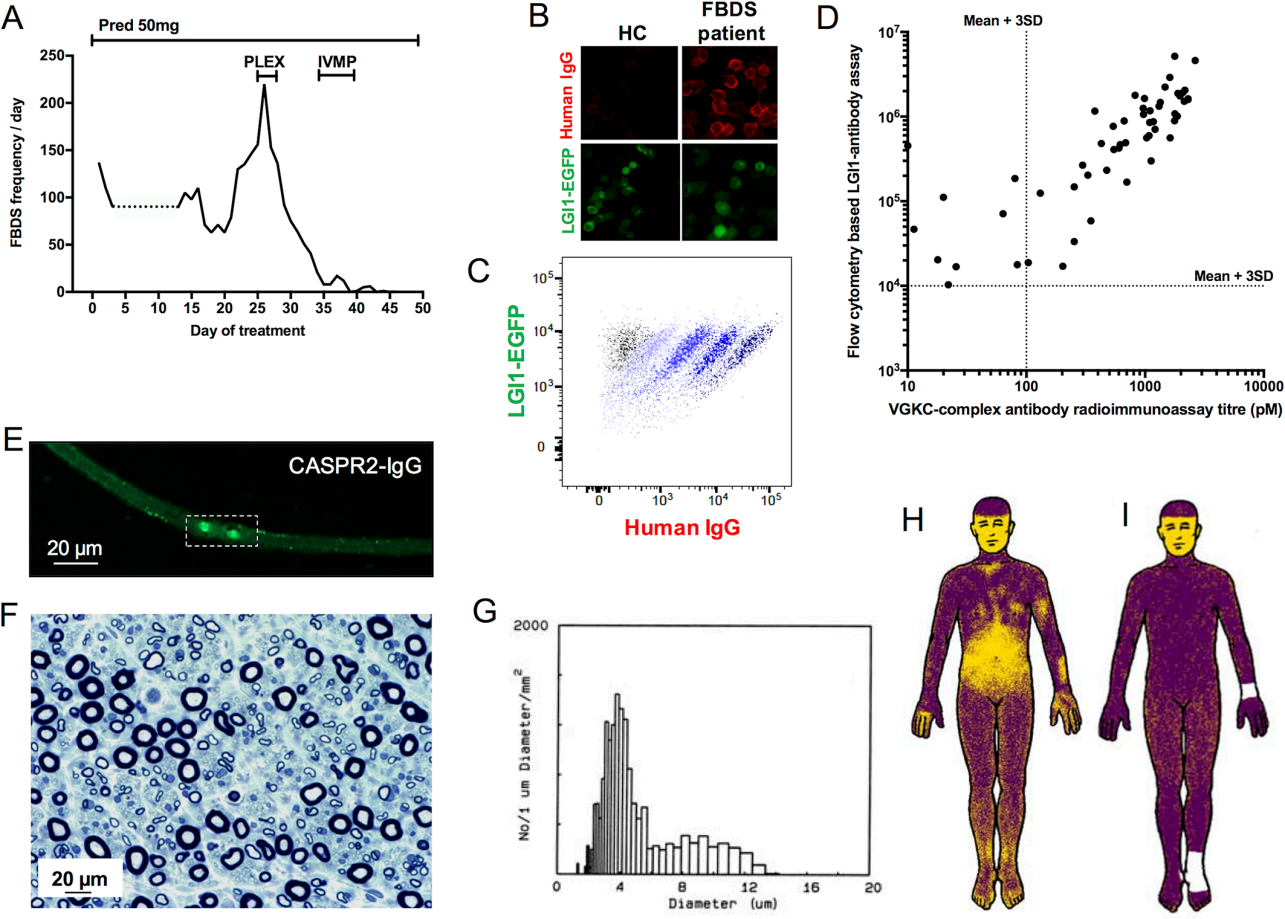
Clinical and serological features of patients with LGI1 and CASPR2 antibodies. (A) Daily frequency of faciobrachial dystonic seizures as recorded prospectively by the patient in figure 1 and the online video. Days 4–12 not recorded by patient (dotted line). His FBDS began with a frequency of 10 per day and rose to 200 per day, with a fall after corticosteroid initiation to around 150 per day. The remainder of his clinical progress is shown in figure 2A and suggests a marked improvement after PLEX. (B) Live LGI1 antibody cell based assay in the patient (from A) with FBDS and a healthy control (HC) showing human IgG deposition on the surface of HEK 293 T cells transfected with membrane-tethered EGFP-tagged LGI1 (LGI1-EGFP). (C) Levels of human IgG bound to surface-expressed LGI1-EGFP may also be quantified by flow cytometry. Five clouds of cells showing increasing IgG binding, left to right. (D) From patients with limbic encephalitis and autoimmune epilepsies, VGKC complex antibody determination misses some patients with LGI1 antibodies proven by flow cytometry. Both cut-offs determined as mean plus 3 SD. (E) CASPR2-IgG deposition in the sural nerve of a patient with peripheral nerve symptoms and CASPR2 antibodies who shows normal sural nerve morphology (F) by morphometric analysis in both large (8–12 µm) and small (<5 µm) nerve fibre density (G). This supports a neurophysiological disruption at the node with potential for reversibility. Thermal sweat test using alizarin red powder; face not tested, loss of sweat in yellow and sweating in blue with normalisation after immunotherapy (panel H compared with panel I. Panels (E–I) reproduced with permissions.^8^ CASPR2, contactin-associated protein 2; EGFP, enhanced green flourescent protein; FBDS, faciobrachial dystonic seizures; IVMP, 3 days of 1 g intravenous methylprednisolone; LGI1, leucine-rich glioma-inactivated 1; Pred, prednisolone; PLEX, plasma exchange; VGKC, voltage-gated potassium channel.

**Figure 3:**
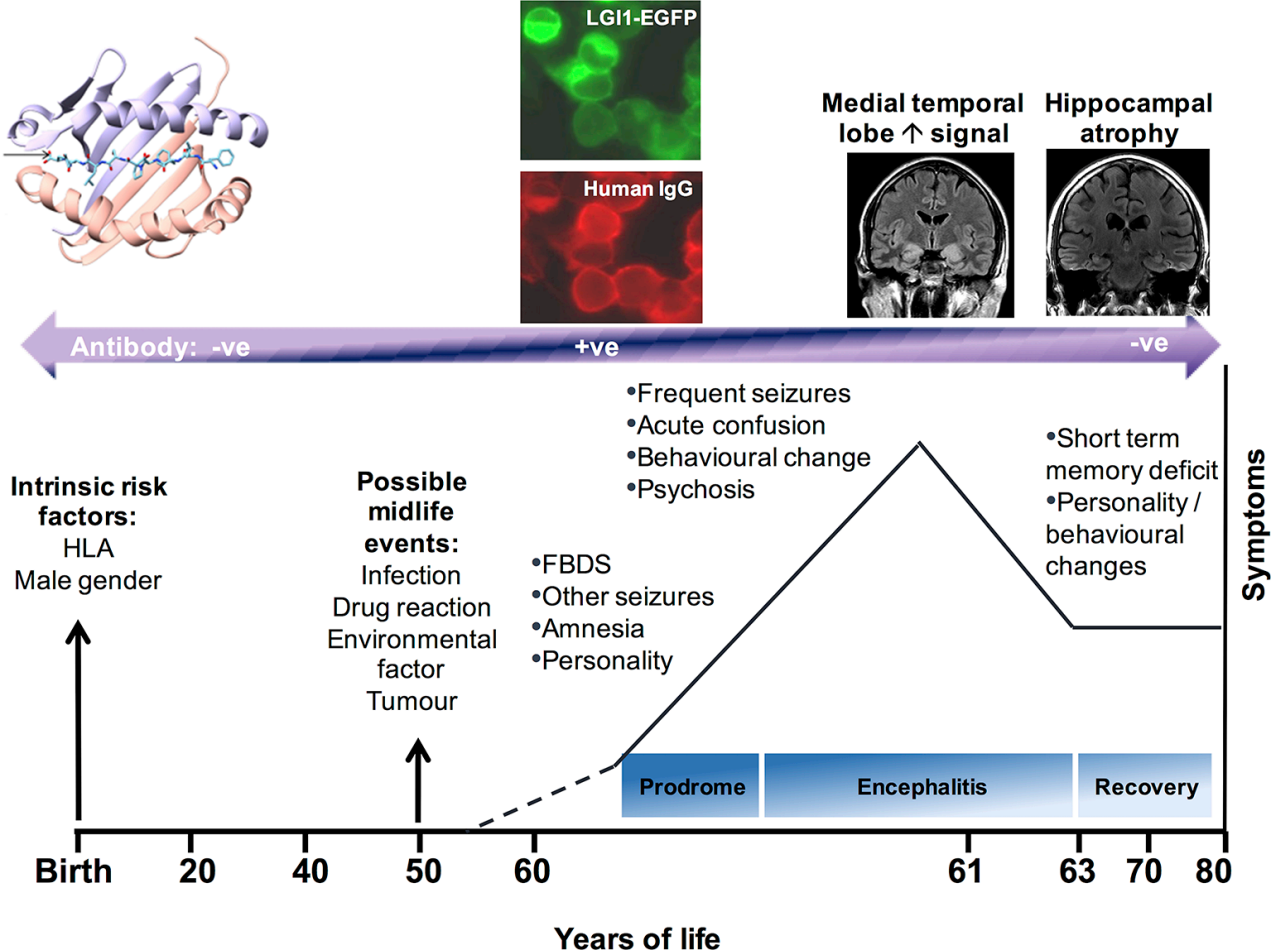
The evolution of LGI1 antibody disease. Schematic diagram to show the likely risk factors for developing LGI1 antibody encephalitis, possible midlife events, followed by the development of antibody positivity and onset of a prodrome of seizures, including FBDS and cognitive alterations. As these worsen into a full-blown encephalitic syndrome, medial temporal lobe hyperintensities are often noted. After immunotherapies, the disease often resolves to leave persistent cognitive deficits and hippocampal atrophy. Human leucocyte antigen (HLA) diagram reproduced with permissions.^41^ EGFP, enhanced green flourescent protein; FBDS, faciobrachial dystonic seizures; LGI1, leucine-rich glioma-inactivated 1.

Frequent focal seizures and amnesia are the two hallmarks of LGI1 and CASPR2 antibody-related central nervous system (CNS) diseases. These, and other aspects of the disease and antibodies, are illustrated in figures 1–3.

### Seizures

I was terrified to do anything, make a cup of tea, or if someone would speak, I would get a violent spasm…I could also get a spasm if I was excited, for example, watching [a football team] I would say ‘please don’t score’.—Impact of FBDS in 59-year-old male patient

Rich hippocampal and limbic system expression of both CASPR2 and LGI1 ensures vulnerability of epileptogenic areas to these autoantibodies.^13^ Indeed, seizures are frequent in both LGI1 and CASPR2 antibody associated syndromes, and isolated epilepsy may be a presentation of both.^20^ Overall, seizures are more common in patients with LGI1 antibodies: in the first comparative study, seizure rates were 90% versus 53% in those with CASPR2 antibodies.^4^ In CASPR2 antibody patients, seizures appear related to the presence of cerebrospinal fluid (CSF) CASPR2 antibodies, which may be less frequent in patients with Morvan’s syndrome or NMT.^21^ While the precise seizure semiologies are not well-reported in patients with CASPR2 antibodies, they appear diagnostic in many cases with LGI1 antibodies. Indeed, the single most striking and robust difference between patients with LGI1 and CASPR2 antibodies is the association of FBDS with LGI1 antibodies. FBDS are brief episodic events that consist of arm and ipsilateral hemiface posturing that last a few seconds and affect, in descending frequency, the arm, face and leg (online supplementary video).^9 15^ They may occur hundreds of times per day at maximum (figure 2A) and affect between 34% and 68% of patients with LGI1 antibodies.^9 22^ Unusual triggers include rapid movements, high emotion, stress and loud noises.^9 23^ They may be preceded by a sensory aura that patients find difficult to crystallise (‘a strange feeling’), and this may offer a clinical clue, as in the earliest stages patients may not notice a jerk and may simply report dropping items or a very subtle hand movement (Binks and Irani, unpublished observations). Moreover, FBDS themselves are not benign, as they are associated with falls and consequent injury.^9 23^ Importantly, FBDS usually precede the cognitive impairment associated with frank limbic encephalitis and are important to recognise as early treatment with immunosuppression appears to abort progression to this highly disabling disease state.^9^

10.1136/jnnp-2017-315720.supp1 Supplementary file 1

I went to A&E with three ‘fainting episodes’. These were reoccurring and accompanied with numbness and tingling in my limbs, goose bumps, change of pallor, flushing, hot and cold sweats and general feeling of nausea…It felt as though I was having an abnormal physiological response to a change in body temperature. It was how you feel when you’ve got a virus or flu, but lasted for less than a minute.—Description of autonomic seizures with piloerection from 61-year-old man with LGI1 antibodies

In addition to FBDS, several other focal seizure semiologies are recognised in patients with LGI1 antibodies and are relatively infrequent in other seizure disorders. These include gelastic seizures, ictal bradycardia and a range of sensory and autonomic seizures, often with piloerection and thermal sensations including ‘shivering’ or ‘flushing’.^10 15 22 24^ Another episodic phenomenon has been termed ‘paroxysmal dizziness spells’, and were recently recognised to be events—likely seizures—that are exclusive to the patients with LGI1 antibodies.^8^ Overall, generalised seizures are far less frequent than focal seizures in both conditions, but status epilepticus may occur, and both antibodies should be tested as part of the work-up in new-onset refractory status epilepticus.^25^

### Amnesia and cognitive disturbance

Woke and memory gone – forgotten years – only very early memories. Totally confused and very, very frightened.—Spousal diary of symptoms affecting 56-year-old male with LGI1 antibodies

Amnesia is the second major hallmark of both antibody groups.^4 8 11 21 22^ Patients often have prominent autobiographical memory loss with marked disorientation and confusion in the acute phase.^4 26^ By the chronic disease stage, there are typically clear improvements in overall cognitive scores but often a focal memory deficit persists with relative preservation of executive functions, attention, language and visuospatial skills.^27^ This residual pattern of amnesia likely relates well to both the relatively focal CA3 hippocampal atrophy and the CNS distribution of LGI1.^22 27 28^

Other neuropsychiatric manifestations include psychosis, with hallucinations a common feature of patients with Morvan’s syndrome who most commonly have coexistence of both LGI1 and CASPR2 antibodies.^13^ Also, personality change and depression are frequently observed.^20^ As these are also sometimes features of patients with dominant epilepsy presentations, one challenge for the field is to better define the concept of ‘isolated autoimmune epilepsy’.

While the cognitive impairment and seizures have traditionally been separately classified, both subclinical and clinical seizures may contribute to the cognitive impairment. This is most likely for FBDS where the cognitive impairment often appears as the seizure frequency reaches a very frequent crescendo.^15^ In addition, one recent video-telemetry study has shown a correlation between frequency of all LGI1 antibody-related seizures and long-term outcomes, implying a potentially persistent effect of seizure frequency on disability.^24^

### Neuromyotonia

My wife noticed my skin jumping while I was sleeping, soon I noticed it while awake and I would get painful muscle cramps in my thighs, legs and feet, I also noticed muscle twitching in these and other muscles.—32-year-old man with CASPR2 antibodies and electrophysiological evidence of NMT

Another relatively specific feature of patients with CASPR2 antibodies in particular is NMT, a form of peripheral nerve hyperexcitability. Patients develop cramps, stiffness, fasciculations and sometimes CNS features including depression and amnesia.^13^ Electromyography findings can include myokymia, fasciculations, as well as classic NMT with spontaneous and continuous motor unit discharges with a high intraburst frequency. Often patients have reduced motor and sensory nerve action potential amplitudes suggesting potential for loss of axons over time. Large complex motor units can be seen with reduced recruitment, characteristic of a motor axononpathy. The presence of isolated NMT, or NMT as part of Morvan’s syndrome, is an important red flag in detailed screening for a thymoma. Studies of plasma exchange (PLEX) and passive antibody transfer to experimental animals provided the earliest evidence that NMT was an antibody-mediated disease, although the antigenic targets were not determined in these historical studies.^7^ The relatively lower rates of NMT in LGI1 antibody-positive patients likely relates to the greater expression of CASPR2 in the periphery. Since acquired NMT is both rare and almost exclusively exists with one or both antibodies, this feature also appears suggestive of a shared functional disease pathway. Yet, it is not known why some patients with serum NSAbs—both of CASPR2 and LGI1 specificities—and accessible peripheral nerve targets, only have CNS features, but a younger age is the only known association of a restricted peripheral nerve presentation.^8^

### Movement disorders

He was shaky in all four limbs…he would be shaking during a meal…he had whole body shakes, and was slow, he was like an 80-year-old with dementia.— Spouse of 51-year-old patient with LGI1 antibodies

Movement disorders are reported at low but appreciable rates in both antibody groups. A common thread, relating to CNS expression of both proteins, may be cerebellar involvement: CASPR2 antibodies have been detected in as many as 10% of patients with ‘idiopathic ataxia’,^29^ while cerebellar features have been identified in 8% of patients harbouring LGI1 antibodies.^4^ Chorea is also an unusual association of both antibodies and has a wide differential diagnosis within autoimmune neurology,^30^ but other movement disorders are more divergent between the two antigenic targets. In LGI1 antibody disease, parkinsonism and limb dystonia are observed,^8 15^ whereas orthostatic myoclonus is more suggestive of CASPR2 antibodies.^31^ Overall, these movement disorders are very rarely seen in isolation, and their presence alongside amnesia or focal seizures should highlight LGI1 or CASPR2 antibodies in the differential diagnosis.

### Pain

The pains in my legs have been worsening over the years with restless legs, causing difficulty with sleeping…[and] sharp pains down my right leg. [These are] very painful, lasting a few minutes. I used to be a jovial person, but I feel my personality has changed because the pain and discomfort gets me down.—67-year-old man with CASPR2 and LGI1 antibodies

Neuropathic pain may be isolated or combined with other clinical features and, importantly, may be immunotherapy responsive.^8^ In both early and more recent studies, neuropathic pain affected around 40% of patients with CASPR2 antibodies compared with 15% of patients with LGI1 antibodies.^4 8^ Like NMT, this may be consistent with dense peripheral nervous system (PNS) expression of CASPR2. The generation of CASPR2 antibody-mediated NMT and neuropathic pain may relate to the functional antibody effects. Indeed, some CASPR2 antibody-positive patients have no loss of fibres on peripheral nerve biopsy, preserved CASPR2 expression and normal thermal sweat testing (figure 2E–G). Other patients have evidence of small fibre peripheral neuropathy as demonstrated by the thermoregulatory sweat test (figure 2H,I). This has also been observed in some LGI1 antibody-positive patients.^32^ These patients may represent a true hyperexcitable disorder (figure 2E). However, some patients also have loss of axonal fibres and reduced amplitudes on nerve conduction studies. The relative roles of complement fixing IgG1-CASPR2 antibodies and non-complement activating IgG4-CASPR2 antibodies should be compared in these two patient groups.

### Dysautonomia and hypothalamic dysfunction

Dysautonomia is a defining feature of Morvan’s syndrome,^13^ typically in patients with CASPR2 antibodies. But is also a feature in patients with both LGI1 and CASPR2 antibody encephalitis, affecting up to 23% in large cohorts.^4 8^ Cardiac complications are potentially life threatening and in both patient groups require clinical vigilance. Severe bradycardia requiring pacemaker insertion is recognised with LGI1 antibodies, tending to predate limbic encephalitis and likely relating to insular or temporal lobe epileptic activity.^33^ Also, asystole, QT-interval prolongation, bradycardia and sudden cardiac death are all recognised in connection with CASPR2 antibodies, particularly in patients with Morvan’s syndrome.^8 13^ Autonomic dysfunction can also manifest with hyperhidrosis, diarrhoea, urinary dysfunction, hypertension and hypotension.^13^

Around 50% of patients with LGI1 antibodies and FBDS are hyponatraemic.^4 6 15^ The mechanism may involve the established capacity of LGI1 antibodies to bind anti-diuretic hormone (ADH)-secreting hypothalamic neurons.^13^ Although hyponatraemia may be seen with CASPR2 antibody positivity, this is typically at significantly lower rates, and the CASPR2 antibodies do not bind the ADH-secreting neurons, suggesting an alternative mechanism is required to explain the observation. Such regional binding may also account for hypothermic attacks in patients with LGI1 antibodies, in which body temperatures as low as 33°C have been recorded.^34^

### Sleep disturbance

Complained of extreme tiredness…dozed off constantly…(next day)…slept 16 hours….[next day] slept 36 hours with breaks for food/drink.—Spouse of 69-year-old man with LGI1 antibodies

Insomnia is a cardinal feature of Morvan’s syndrome and can associate with reversed diurnal rhythms and the syndrome of agrypnia excitata.^13^ Morvan’s syndrome is rarer in patients with only LGI1 antibodies and, indeed, CASPR2 antibodies often associate more frequently with insomnia and agrypnia.^8^ Aside from Morvan’s syndrome, insomnia is reported in patients with LGI1 antibodies at rates of 7%–23%.^4 8 23^ The total number of patients affected by any sleep disturbance, including hypersomnolence and rapid eye movement sleep disorder as well as insomnia, reaches 31%–48%. The sleep disorders appear to improve with immunotherapies. Expression of CASPR2 and LGI1 in both raphe nuclei and thalamus may explain the prominence of sleep disorders in patients with antibodies directed against these antigens.^13^

## The paraclinical features

### Electroencephalography (EEG) and MRI

EEG may be normal in both antibody groups, and a normal EEG should not deter from the diagnosis in the appropriate setting.^4 8 15^ Although many patients with FBDS, sensory or autonomic seizures have normal EEGs during events, frontocentral cortical slow waves have been reported preceding and contralateral to the FBDS.^23^ In addition, subclinical seizures are frequent in these patients,^10 15 23 24^ and this adds weight to the notion that seizures are important contributors to the underlying cognitive deficits.

The brain MRI in these patients is normal in around 50% of cases, and the diagnosis should not be overlooked in the absence of the, now iconic, unilateral or bilateral hippocampal swelling with hyperintensities on T2 and FLAIR sequences.^4 6 8^ These have been reported to sometimes show contrast enhancement and diffusion restriction in the acute phase.^12^ In addition, patients with FBDS often show basal ganglia changes in T2, plus T1, -weighted imaging. The latter may be a specific biomarker for FBDS.^8 9 35^ Areas of swelling often resolve to leave atrophy, which—if hippocampal—can subsequently manifest as adult-onset medial temporal lobe epilepsy.^36^ Whether the presence of swelling correlates with the antibody subclass, and the potential for complement fixation or lymphocyte recruitment, is unknown but these data could help target more aggressive therapies at patients with the potential for hippocampal atrophy. Although it seems unlikely that the established hippocampal atrophy might respond to immunotherapy, further studies may investigate broader immune contributions to this residual damage.

### Antibody: subtype, synthesis and specificities

A key shared feature of both antibody groups is the high prevalence of antigen-specific antibodies of the IgG4 subclass.^11 13 21^ As discussed above, IgG4 antibodies do not fix complement but may act through direct modulation of their antigenic target. However, available neuropathology reports have demonstrated neuroinflammatory changes, which may be a result of the IgG1 antibodies, the second most commonly detected subclass.^37^ In one study, patients with CASPR2 antibodies and subsequent hippocampal atrophy were all shown to harbour IgG1 antibodies,^21^ and our recent observations suggest that higher levels of serum IgG1 LGI1 antibodies correlate with increasing cognitive impairment (Makuch and Irani, unpublished). Indeed, this chronic cognitive impairment is a very common sequela of LGI1 and CASPR2 antibody encephalitis and should be a focus of future research.

Most studies suggest that presence of serum LGI1 or CASPR2 antibodies is more sensitive than CSF determination,^8 10 13^ and only one study suggests CSF testing is more sensitive.^11^ Furthermore, in addition to avoidance of positive results in patients with non-immune conditions,^5^ serum testing with specific cell-based assays (CBAs) for CASPR2 and LGI1 antibodies surpasses the sensitivity of VGKC complex antibody determination (figure 2B–D).^8 9 29^ Therefore, these are two compelling reasons to halt future VGKC complex antibody testing in routine practice. However, fixed CBAs for CASPR2 antibodies have revealed a low-level positivity rate in healthy controls or non-disease relevant settings,^38^ and it remains important to interpret results alongside appropriate clinical information. Using live CBAs, we have also detected CASPR2 antibodies in clinically irrelevant situations (Binks and Irani, in submission). However, the LGI1 antibody live CBA typically shows excellent specificity, and detection is comparable in accuracy to the more quantitative, and higher throughput, detection with flow cytometry (figure 2C). The value of sequential titres is not yet known, although it is well established that antibodies may persist for at least 2 years postclinical recovery^22^ and much longer for some patients (Binks and Irani, unpublished). The clinical relevance of these persistent antibodies seems limited as, in our experience, patients can have rapid recoveries with long-lasting antibodies and vice versa.

## Treatments

### Immunotherapies

A practical and empirical approach to treatment is required, as the limited available data are largely retrospective and observational. Across all LGI1 and CASPR2 antibody associated syndromes, immunotherapies are the mainstay of treatment, and immunotherapy escalation is determined by clinical evidence of improvement. Particularly in LGI1-associated FBDS and other focal seizures, there is clear evidence of often dramatic seizure cessation with corticosteroids, which appear relatively resistant to antiepileptic drugs (AEDs).^9 10 15^ Crucially, this rapid impact may mediate the effect of early immunotherapy in the possible prevention of disease progression to limbic encephalitis. CASPR2 antibody-associated seizures have been reported as less responsive to immunotherapy and, in a comprehensive study, 75% required at least two types of immunotherapy and a median of 14 months until seizure control.^38^

The most frequently used additional immunotherapies are intravenous immunoglobulins (IVIG) and PLEX which, combined with corticosteroids, lead to some clinical improvements in almost all patients with LGI1 antibody patients.^4 8 10 11^ However, available data suggest that combination therapy with three or two of these agents, versus corticosteroids alone, does not result in improved outcomes.^1^ Results in patients with CASPR2 antibodies are more mixed but generally show similar improvements.^21 38^ Differences in times to treatment, concomitant supportive therapies and clinical presentation (eg, Morvan’s syndrome or limbic encephalitis) between cohorts are all reasons to support more systematic treatment studies. Indeed, a randomised placebo-controlled trial of IVIG in LGI1 and CASPR2 antibody associated autoimmune epilepsy is currently underway at the Mayo Clinic.

Immunoadsorption, a therapeutic modality for antibody removal, has shown promise perhaps due to its surprising effect on CSF antibodies. A retrospective study showed an expected clinical benefit and fall in serum antibody levels in both CASPR2 and LGI1 antibody patients but also observed very intriguing, and sometimes marked, reductions in CSF antibody levels at early and long-term follow-up.^39^ The depletion of CSF antibodies may explain the often rapid clinical effect of PLEX-based therapies observed in everyday practice. Nevertheless, such improvements are more notable for seizures than for cognition, and this remains an area of unmet clinical need with its associated, and often marked, disability.^9 22^

Other second-line immunotherapies such as azathioprine, mycophenolate and cyclophosphamide may be trialled but have very little supportive observational evidence. It is unclear whether steroid sparing agents have a role to play in diseases, due to their typically prolonged time to action. Perhaps they will be useful to prevent relapses. Particular interest surrounds rituximab (anti-CD20 monoclonal antibody), an agent of utility in IgG4-mediated diseases. So far, data are largely limited to equivocal clinical benefit observed in small case series,^40^ and individual cases within larger cohorts.^8 10 11 15^ However, early anti-CD20 initiation studies, potentially prior to formation of longer lived plasma cells, have not yet been reported.

### Relapses

In the non-paraneoplastic setting, both antibody groups tend to follow a monophasic course, although relapse is reported at highly variable rates, from 14% to 40%.^9–11 15 21 38^ Our clinical experience, and a few reports, suggest that relapses occur during more rapid corticosteroid tapers.^8 9^ In our practice, early and relatively assertive corticosteroids with PLEX, followed by gradual corticosteroid taper over around 18 months, can almost negate relapses. However, at present, our knowledge of biomarkers that predict relapse remains intuitive rather than evidence based.

## LGI1 and CASPR2: the molecular pathology

Differences and similarities are also notable in the emerging, although early and limited, data with respect to disease aetiology. Both antibody groups share evidence of immune dysfunction and a similar subacute time course (figure 3), indicative of mechanistic commonalities.

### Cellular immunology

Recently, LGI1 antibodies have been linked with strong class II human leucocyte antigen (HLA) associations.^41 42^ HLA-DRB1*07:01 was described in around 90% of patients from cohorts derived from East Asian and Caucasian ethnicities. An opportunity to study relevant disease triggers is provided by the known role of HLA class II molecules in presenting exogenously derived antigen to T cells. Furthermore, we are aware of no reports of Afro-Caribbean heritage patients in the LGI1 antibody literature and of very few personally. These ethnic biases may reflect important genetic contributions to the immunology. With similar mechanistic implications, CASPR2 antibody encephalitis has been described secondary to immune system manipulation in the setting of checkpoint inhibitor therapy for melanoma.^43^ These checkpoint inhibitor drugs release T cell regulation of the immune system. If the disinhibited T cells were regulating a population of autoreactive B cells, an autoantibody-mediated disease may arise. Hence, a T–B cell collaboration is likely in the generation of CASPR2 autoimmunity. Indeed, clinically, appreciable rates of coexistent autoimmune disorders have been observed in patients with LGI1 antibodies,^14^ and several patients with CASPR2 antibody have been reported to have both thymomas and myasthenia gravis.^13^ Taken together, these observations hint that more than one ‘hit’ may be required to generate disease: an underlying immunological profile, combined with a predisposing event or antigenic exposure (figure 3). From an immunological perspective, it is also interesting to speculate on why LGI1 and CASPR2 antibodies coexist more often than by chance. Perhaps, a common insult or immunological predisposition leads to epitope spread and preferential presentation of the neighbouring VGKC complexed antigen.

### Human versus animals: genetic and autoimmune models of disease

#### LGI1: temporal lobe and telephones

I was terrified to answer the phone as it would bring a spasm on….the cable would shoot up in the air and the phone drop on my head.—59-year-old male patient with LGI1 antibodies

Animal models and genetic mutations in humans provide valuable opportunities to understand the molecular and clinical features of LGI1 and CASPR2 autoimmunity. Knockout of LGI1 in mice causes lethal myoclonic epilepsy in homozygotes and reduces the seizure threshold in heterozygotes. The myoclonic phenotype might resemble FBDS in human autoimmune patients.^44^ However, unlike patients with LGI1 antibody encephalitis, FBDS are not reported in patients with human mutations of the LGI1 gene, which causes autosomal dominant lateral temporal lobe epilepsy with auditory hallucinations.^45^ Nonetheless, some interesting similarities in seizure semiology do exist between the groups. Both are susceptible to temporal lobe epilepsy and, perhaps even more intriguing is the shared vulnerability to reflex seizures triggered by loud noises, including telephone ringing.^15 46^ This overlap might relate to the neuroanatomical distribution of LGI1 in the temporal lobe or represent common molecular mechanisms secondary to loss of LGI1 function. For example, we have recently discovered that complexes of LGI1 and a disintegrin and metalloprotease 22 (ADAM22; a receptor of LGI1) can be internalised by LGI1 antibodies (Makuch and Irani, unpublished); perhaps a similar mechanism also operates in genetic LGI1 mutants whose phenotypes overlap with their autoimmune counterparts. Alternatively, maybe LGI1 modulation can cause downstream effects via dendrotoxin-sensitive VGKCs^47^ or via functional alterations in AMPA receptors.^28^ A more contrasting phenotypic aspect of humans with genetic versus autoimmune ‘LGI1-opathies’ is their relative responsiveness to AEDs, with the former often requiring only one AED for seizure control. The contrasting developmental versus late-life acquired nature of these diseases may account for the differences, with divergent opportunities for compensatory mechanisms and plasticity governing the relative outputs of the neural networks. These comparative questions are worthy of further study to provide basic insights into early-life versus later-life modulation of the nervous system.

#### CASPR2: neurodevelopment and gender bias

CASPR2, which is coded by the CNTNAP2 gene on chromosome 7, appears to have a role in neurodevelopment and in the correct placement of VGKCs at the tightly defined juxtaparanodes of myelinated axons in the CNS and PNS. In addition, CASPR2 indirectly interacts with ADAM22.^48^ CNTNAP2 knockout mice display abnormal neuronal migration, seizures and an autistic phenotype, features that are often recapitulated in paediatric patients with homozygous CNTNAP2 mutations.^49^ A similar effect may be reflected by CASPR2 antibodies in mothers of children with autism, who might receive in utero exposure, although antibody levels were low in this study.^50^ Moreover, CNTNAP2 mutation-related phenotypes are more frequent in males,^51^ and there is an extremely marked preponderance of males in CASPR2 antibody disease,^13 21 38^ suggesting sex-linked susceptibility to perturbation in CASPR2 function. However, there are also marked differences. For example, while NMT is a frequent peripheral nerve phenomenon in patients with CASPR2 antibodies, surprisingly few peripheral manifestations have been observed in patients with genetic abolition of CNTNAP2, with the exception of reduced deep tendon reflexes.^52^ While, interestingly, in the brain of one patient with a CNTNAP2 mutation, VGKC expression was disrupted,^52^ in the murine genetic CNTNAP2 knockouts, dispersion of juxtaparanodal VGKCs did not translate to measurable effects on VGKC currents. Yet, the effect of passive transfer of IgG from patients with NMT was electrophysiologically consistent with VGKC disruption.^53^ This may be related to modulation of juxtaparanodal molecules that consistently associate with CASPR2, such as contactin-2.^3^ Therefore, again a full understanding of the relative differences in mechanisms between genetic and antibody-mediated disease models of molecular disruption may offer explanations for the differential manifestations in LGI1-mediated and CASPR2-mediated diseases, as well as other analogous autoimmune scenarios.^3^ Data from the genetic and autoimmune models of CASPR2 disruption may yet suggest the VGKCs are a common effector pathway.

## Conclusions

This review has highlighted both the shared and distinct features of LGI1 and CASPR2 antibody diseases. Data on antigenic targets are scientifically robust, and direct testing for LGI1 and CASPR2 antibodies has marked clinical advantages. Yet the striking overlapping disease spectrum, the existence of both antibodies in ‘double-positive’ patients and the synchronous discovery of these targets should not be easily dismissed from a mechanistic viewpoint. Indeed, deeper links are ripe for exploration, and a unifying role for VGKCs merits consideration, since a central physiological function of both proteins is found in their relationship to VGKCs, in addition to ADAM22. Thus, the proteins are operationally distinct but functionally linked through their mutual association with VGKCs: a pattern that is mimicked at the patient level. We therefore consider the field to be moving towards a molecular *evolution*, not a molecular *revolution*.
